# Genome wide association study and genomic prediction for fatty acid composition in Chinese Simmental beef cattle using high density SNP array

**DOI:** 10.1186/s12864-017-3847-7

**Published:** 2017-06-14

**Authors:** Bo Zhu, Hong Niu, Wengang Zhang, Zezhao Wang, Yonghu Liang, Long Guan, Peng Guo, Yan Chen, Lupei Zhang, Yong Guo, Heming Ni, Xue Gao, Huijiang Gao, Lingyang Xu, Junya Li

**Affiliations:** 1 Laboratory of Molecular Biology and Bovine Breeding, Institute of Animal Sciences, Chinese Academy of Agricultural Sciences, Beijing, China; 2 Animal Science and Technology College, Beijing University of Agriculture, Beijing, China; 3 Haidian District, Institute of Animal Science, Yuanmingyuan West Road 2#, Beijing, 100193 China

**Keywords:** Fatty acid, Genome-wide association study, Genomic prediction, High-density genotypes, Simmental cattle

## Abstract

**Background:**

Fatty acid composition of muscle is an important trait contributing to meat quality. Recently, genome-wide association study (GWAS) has been extensively used to explore the molecular mechanism underlying important traits in cattle. In this study, we performed GWAS using high density SNP array to analyze the association between SNPs and fatty acids and evaluated the accuracy of genomic prediction for fatty acids in Chinese Simmental cattle.

**Results:**

Using the BayesB method, we identified 35 and 7 regions in Chinese Simmental cattle that displayed significant associations with individual fatty acids and fatty acid groups, respectively. We further obtained several candidate genes which may be involved in fatty acid biosynthesis including elongation of very long chain fatty acids protein 5 (*ELOVL5*), fatty acid synthase (*FASN*), caspase 2 (*CASP2*) and thyroglobulin (*TG*). Specifically, we obtained strong evidence of association signals for one SNP located at 51.3 Mb for *FASN* using Genome-wide Rapid Association Mixed Model and Regression-Genomic Control (GRAMMAR-GC) approaches. Also, region-based association test identified multiple SNPs within *FASN* and *ELOVL5* for C14:0. In addition, our result revealed that the effectiveness of genomic prediction for fatty acid composition using BayesB was slightly superior over GBLUP in Chinese Simmental cattle.

**Conclusions:**

We identified several significantly associated regions and loci which can be considered as potential candidate markers for genomics-assisted breeding programs. Using multiple methods, our results revealed that *FASN* and *ELOVL5* are associated with fatty acids with strong evidence. Our finding also suggested that it is feasible to perform genomic selection for fatty acids in Chinese Simmental cattle.

**Electronic supplementary material:**

The online version of this article (doi:10.1186/s12864-017-3847-7) contains supplementary material, which is available to authorized users.

## Background

Fatty acids are required by daily normal metabolism, and can be obtained from food and meat. Improving nutritional value of meat products for human health has attracted extensive attention in current society [1, 2]. Fat content and fatty acid composition in beef products are associated with meat taste and flavor, and these are considered as main sensory properties in consumer’s selection and acceptance [3].

Fatty acids are important indicators of beef meat quality, and previous studies have been conducted to examine fatty acids for various cattle breeds in different feeding environments [4]. Fatty acid composition are often lowly or moderately heritable traits in various populations with different genetic architecture [5]. Several studies have revealed that the level of heritability and genetic correlation theoretically allow for genetic improvement of fatty acid composition by selection of both major candidate genes and genomic selection strategies [6–10]. Therefore, application of molecular genetics approaches can provide an opportunity for genetic improvement for fatty acid composition of beef cattle [11–13].

During the last decades, tremendous works have been done to elucidate the genetic mechanism of fatty acids using candidate gene [14–21] and linkage mapping approaches [22–24]. In recent years, genome-wide association studies (GWAS) have been widely used to study the molecular mechanism underlying important traits in beef and dairy cattle [25–29]. Previous GWAS and genomic predictions have identified candidate markers associated with various fatty acid composition and evaluated the accuracy of genomic prediction for these traits [8, 30–34]. However, many studies were conducted in populations with relatively low density SNP arrays. Despite recent GWAS for fatty acid composition have been investigated using the high density BovineHD (770 K) SNP array, those studies were mostly limited in Nellore cattle [35, 36]. On the other hand, extensive attention has been paid to investigate the accuracies of genomic prediction using multiple methods in different populations [32, 33]. A recent study in Nellore cattle indicated that the accuracies of genomic prediction were moderate to high and it was feasible to apply genomic selection in cattle. However, their results were limited to carcass traits in Nellore population [37]. Therefore, understanding the molecular mechanisms underlying fatty acid composition and evaluating the accuracy of genomic predictions in other important cattle breeds still need further investigation.

The objectives of the current study were to explore the associated genomic regions and estimate the predictive accuracies for fatty acid composition using the BovineHD SNP array in Chinese Simmental population. In this study, we identified several potential candidate markers and genes associated with fatty acid composition. Our findings will facilitate the elucidation of the molecular mechanism and help us design optimal genomic selection strategies for fatty acid composition in cattle and other farm animals.

## Results

### Descriptive statistics of fatty acid composition and their estimates of heritability

We measured six saturated fatty acids (SFA), four monounsaturated fatty acids (MUFA) and eleven polyunsaturated fatty acids (PUFA) using gas chromatography. Descriptive statistics and estimates of heritability for 21 individual fatty acids were presented in Table 1. We observed that the most abundant individual saturated fatty acids were C16:0 (23.8%) and C18:0 (20.2%), while for monounsaturated and polyunsaturated fatty acids, relatively high proportions of individual fatty acids were C18:1 cis-9 (32.0%) and C18:2 n-6 (12.9%). In contrast, we found saturated fatty acids (C20:0, C22:0, C24:0), monounsaturated fatty acids (C14:1 cis-9 and C20:1 cis-11), and polyunsaturated fatty acids (C18:2 t-9c-11, C18:2 t-12c-10, C18:3 n-6, C18:3 n-3, C20:2 n-6, C20:4 n-6, C20:5 n-3, C22:5 n-3, C22:6 n-3) accounted for relatively low proportion (<1% each) of the total fatty acids. In this study, our results found the estimated heritability varied noticeably for these fatty acids. Among 21 individual fatty acids, we found only C14:0 showed a relatively high heritability at 0.54 and five fatty acids including C18:0, C20:0, C14:1 cis-9, C16:1 cis-9 and C18:1 cis-9 showed a moderate heritability, while most of heritability estimates for others fatty acids (15 out of 21) were below 0.2 (Table 1). For the eight groups of fatty acids, we found MUFA, n-6/n-3 and health index (HI) showed moderate heritabilities (0.27, 0.22 and 0.24), while the estimated heritability for SFA, PUFA, total of omega 3 (n-3), total of omega 6 (n-6) were 0.12, 0.16, 0.15, and 0.16, respectively.

**Table 1:** Summary statistics of mean (%), standard deviation (SD, %) and heritability estimates (*h* ^*2*^), additive genetic variance and coefficient of variation (CV%)

Trait^a^	Name	Mean ± SD	Additive genetic variance	Residual variance	CV%	h^2^ ± SE
IMF	Intramuscular fat	4.09 ± 2.24	0.8132	3.4668	54.77	0.19 ± 0.10
Saturated						
C14:0	Myristic	1.58 ± 0.45	0.0694	0.0599	28.48	0.54 ± 0.14
C16:0	Palmitic	23.79 ± 8.75	4.5292	43.1737	36.78	0.09 ± 0.04
C18:0	Stearic	20.23 ± 3.47	2.4048	4.5660	17.15	0.34 ± 0.12
C20:0	Arachidic	0.38 ± 0.29	0.0397	0.1257	76.32	0.24 ± 0.06
C22:0	Behenic	0.13 ± 0.12	0.0009	0.0109	92.31	0.08 ± 0.07
C24:0	Methyl tetracosanoate	0.36 ± 0.17	0.0014	0.0275	47.22	0.05 ± 0.06
Monounsaturated						
C14:1 cis-9	Methyl myristoleate	0.41 ± 0.24	0.0101	0.0373	58.54	0.21 ± 0.08
C16:1 cis-9	Methyl palmitoleate	2.08 ± 1.07	0.2100	0.6590	51.44	0.24 ± 0.07
C18:1 cis-9	Oleic	31.98 ± 6.69	4.9380	16.6498	20.92	0.23 ± 0.09
C20:1 cis-11	Cis-11-eicosenoic acid methyl ester	0.26 ± 0.13	0.0008	0.0161	48.16	0.05 ± 0.07
Polyunsaturated						
C18:2 n-6	Linoleic	12.93 ± 5.84	3.8965	18.2536	45.17	0.18 ± 0.10
C18:2 t-9c-11	cis-9 and trans-11 octadecadienoic acid methyl esters	0.42 ± 0.27	0.0065	0.0663	63.15	0.09 ± 0.08
C18:2 t-12c-10	cis-10 and trans-12 octadecadienoic acid methyl esters	0.16 ± 0.13	0.0009	0.0161	81.25	0.05 ± 0.04
C18:3 n-6	Methyl γ-linoleate	0.41 ± 0.32	0.0054	0.0630	78.05	0.08 ± 0.05
C18:3 n-3	Linolenic	0.68 ± 0.32	0.0085	0.0711	47.06	0.11 ± 0.06
C20:2 n-6	Cis-11,14-eicosadienoic acid methyl ester	0.17 ± 0.13	0.0132	0.1522	75.76	0.08 ± 0.05
C20:3 n-3	Cis-11,14,17-eicosatrienoic acid methyl ester	2.75 ± 1.42	0.2328	1.2310	51.64	0.16 ± 0.08
C20:4 n-6	Cis-5,8,11,14-eicosatetraenoic acid methyl ester	0.03 ± 0.02	2.4e-5	3.76e-4	66.67	0.06 ± 0.04
C20:5 n-3	Cis-5,8,11,14,17-eicosapentaenoic acid methyl ester	0.32 ± 0.21	0.0036	0.0233	65.63	0.13 ± 0.06
C22:5 n-3	Cis-7,10,13,16,19-docosapentaenoic methyl ester	0.73 ± 0.43	0.0240	0.1609	58.85	0.13 ± 0.07
C22:6 n-3	Cis-4,7,10,13,16,19-docosahexaenoic acid methyl ester	0.20 ± 0.18	0.0013	0.0311	93.20	0.04 ± 0.06
Fatty acid groups						
SFA	Sum of saturated fatty acid	46.47 ± 8.23	6.9372	50.8728	17.71	0.12 ± 0.08
MUFA	Sum of monounsaturated fatty acid	34.73 ± 6.97	6.1980	16.7576	20.07	0.27 ± 0.10
PUFA	Sum of polyunsaturated fatty acid	18.8 ± 8.72	7.5442	39.6071	46.38	0.16 ± 0.09
PUFA/SFA	Ration between PUFA and SFA	0.4 ± 0.23	0.0092	0.04249	56.48	0.18 ± 0.09
n-3	Sum of omega 3 fatty acids	4.68 ± 2.64	0.7182	4.0698	56.41	0.15 ± 0.08
n-6	Sum of omega 6 fatty acids	13.54 ± 6.28	3.8914	20.4299	46.38	0.16 ± 0.08
n-6/n-3	Ratio between n-6 and n-3 PUFA	2.89 ± 0.64	0.074	0.257	22.21	0.22 ± 0.11
HI	Health index	1.78 ± 0.37	0.2894	0.9165	20.83	0.24 ± 0.10

^a^The concentrations of fatty acids were expressed as a percentage of total fatty acid methyl esters quantified

### Phenotypic and genetic correlations

**Fig. 1:**
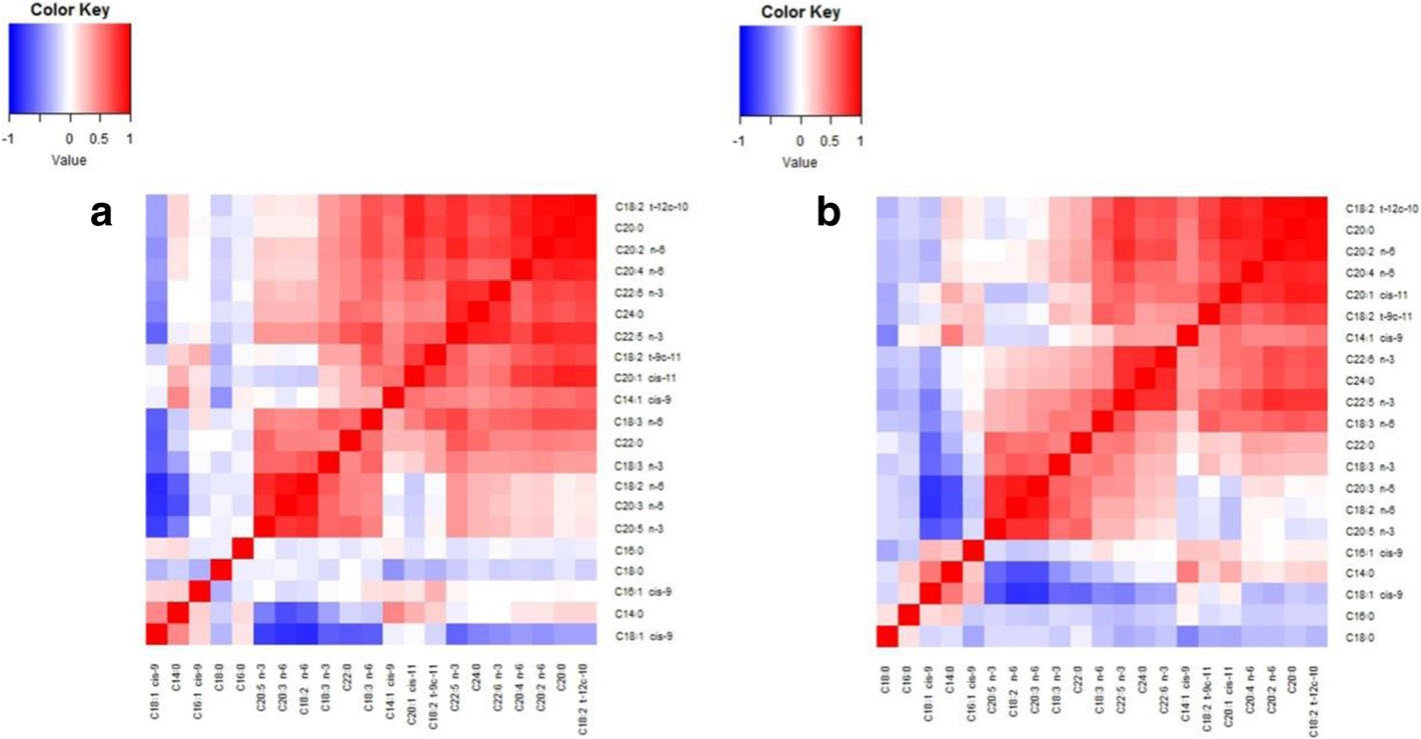
Heatmap of phenotypic (**a**) and genetic correlation (**b**) across 21 individual fatty acid compositions

Phenotypic and genetic correlations among 21 individual fatty acids were presented in Fig. 1. The estimated phenotype correlation of these fatty acids (Fig. 1a) generally displayed different patterns compared to genetic counterparts (Fig. 1b). We observed that high positive phenotypic and genetic correlations existed between several pairs of individual fatty acids. For instance, the estimated genetic correlations between C20:0 and C20:1 cis-11, C20:0 and C18:2 t-12c-10, C20:0 and C20:2 n-6 and C20:0 and C20:4 n-6 were 0.89, 0.95, 0.92 and 0.86, respectively. In contrast, we found clear negative correlations between C14:0 and C18:2 n-6, C14:0 and C20:3 n-3, C18:1 cis-9 and C18:2 n-6, C18:1 cis-9 and C20:5 n-3 (Additional file 1: Table S1).

### Bayesian based GWAS and candidate regions

We performed GWAS using the BayesB method for 11 individual fatty acids that showed estimated genomic heritability ≥ 0.10, including 3 saturated fatty acids (C14:0, C18:0 and C20:0), 3 monounsaturated fatty acids (C14:1 cis-9, C16:1 cis-9 and C18:1 cis-9) and 5 polyunsaturated fatty acids (C18:2 n-6, C18:3 n-3, C20:3 n-3, C20:5 n-3 and C22:5 n-3). To identify potential regions associated with fatty acids, we divided the genome into 100 kb windows, leading to 24,900 regions across the genome. Regions that explain more than 1% of additive genetic variances were considered as candidates and subject to further analyses to identify the associated genes within these regions. Summary statistics including genetic variances explained, position for the 100 kb windows, flanking rs number ID for these regions, and candidate genes of saturated fatty acid, monounsaturated fatty acid and polyunsaturated fatty acids were represented in Tables 2 and 3, respectively.

**Table 2:** Genomic regions associated with the saturated fatty acids in Chinese Simmental cattle using BayesB method

FA Trait	Chr	Associated Window	nSNP	rs_ID_start	rs_ID_end	Genetic variance for 100 kb window	Positional/putative candidate gene	Gene name
C14:0	chr25	15100001:15200000	37	rs134854144	rs136080067	0.014154507		
	chr23	25100001:25200000	47	rs132630789	rs109109903	0.014619034	*ELOVL5*	Bos taurus Elongation of very long chain fatty acids protein 5
	chr22	59100001:59200000	47	rs41638475	rs109645438	0.074590648	*HDAC11* *NUP210*	Bos taurus histone deacetylase 11 Bos taurus nucleoporin 210
	chr19	51300001:51400000	22	rs41921222	rs41919992	0.100435511	*FASN*	Bos taurus fatty acid synthase
C18:0	chr6	104300001:104400000	37	rs43482940	rs134151071	0.011996761	*DMP1*	Bos taurus dentin matrix acidic phosphoprotein 1
	chr6	70900001:71000000	33	rs108998288	rs137260120	0.012677451		
	chr6	41100001:41200000	19	rs134035533	rs43461220	0.018256368	*SLIT2*	Bos taurus slit guidance ligand 2
C20:0	chr2	11700001:11800000	31	rs134430461	rs109568935	0.028998035		
	chr4	107500001:107600000	23	rs132935788	rs135713587	0.012116165	*CASP2* *CLCN1* *FAM131B* *ZYX*	Bos taurus caspase 2, apoptosis-related cysteine peptidase Bos taurus chloride voltage-gated channel 1 Bos taurus family with sequence similarity 131 member B Bos taurus zyxin
	chr6	24100001:24200000	20	rs110959363	rs110137781	0.018578393		
	chr7	111100001:111200000	31	rs133722129	rs43539829	0.013985525	*PJA2*	Bos taurus praja ring finger 2, E3 ubiquitin protein ligase
	chr12	13800001:13900000	36	rs132809847	rs109642481	0.012955962		
	chr12	71200001:71300000	2	rs137175740	rs42873431	0.022999321		
	chr14	35500001:35600000	24	rs136365597	rs137334521	0.062770091		
	chr15	47400001:47500000	16	rs42670335	rs41772249	0.032833294	*FAM160A2*	Bos taurus family with sequence similarity 160 member A2
	chr23	11400001:11500000	16	rs133114050	rs110729743	0.015925179

**Table 3:** Genomic regions associated with the monounsaturated and polyunsaturated fatty acids in Chinese Simmental cattle using BayesB method

FA Trait	Chr	Associated Window	nSNP	rs_ID_start	rs_ID_end	Genetic variance for 100 kb window	Positional/putative candidate gene	Gene name
C14:1 cis-9	chr2	14700001:14800000	24	rs109872420	rs42238053	0.016989227	*SSFA2*	Bos taurus sperm specific antigen 2
	chr19	51300001:51400000	22	rs41921222	rs41919992	0.064947269	*FASN*	Bos taurus fatty acid synthase
	chr22	40200001:40300000	50	rs43704022	rs134738297	0.012042417	*PTPRG*	Bos taurus protein tyrosine phosphatase, receptor type, G
C16:1 cis-9	chr5	62300001:62400000	23	rs109883934	rs135394103	0.023992534		
	chr14	9400001:9500000	21	rs110610151	rs133268603	0.039269923	*TG*	Bos taurus thyroglobulin
C18:1 cis-9	chr12	76800001:76900000	43	rs42705033	rs133235260	0.013243828	*CLDN10* *DZIP1*	Bos taurus claudin 10 Bos taurus DAZ interacting zinc finger protein 1
	chr14	30100001:30200000	14	rs133107941	rs109885660	0.022457664		
	chr14	30300001:30400000	27	rs135047094	rs41733749	0.194380284		
C18:2 n-6	chr4	83500001:83600000	34	rs42446310	rs42501954	0.023282701	*LSM8*	Bos taurus LSM8 homolog, U6 small nuclear RNA associated
	chr20	23500001:23600000	14	rs109459626	rs110757204	0.049524271	*SLC38A9*	Bos taurus solute carrier family 38 member 9
C18:3 n-3 C20:3 n-3	chr14	30300001:30400000	27	rs135047094	rs41733749	0.028254641		
	chr4	83500001:83600000	34	rs42446310	rs42501954	0.010587293	*LSM8*	Bos taurus LSM8 homolog, U6 small nuclear RNA associated
	chr17	68400001:68500000	27	rs110865754	rs136952413	0.017868447	*TFIP11* *TPST2*	Bos taurus tuftelin interacting protein 11 Bos taurus tyrosylprotein sulfotransferase 2
C20:5 n-3	chr3	55400001:55500000	27	rs137055781	rs135699972	0.017277515		
	chr5	110800001:110900000	35	rs41593844	rs109225081	0.010709916	*CBY1* *TOMM22* *JOSD1* *GTPBP1* *SUN2*	Bos taurus chibby homolog 1 (Drosophila). Bos taurus translocase of outer mitochondrial membrane 22 Bos taurus Josephin domain containing 1 Bos taurus GTP binding protein 1 Bos taurus Sad1 and UNC84 domain containing 2
	chr12	22800001:22900000	42	rs134078558	rs135218498	0.014090239	*LHFP*	Bos taurus lipoma HMGIC fusion partner
C22:5 n-3	chr2	11700001:11800000	31	rs134430461	rs109568935	0.012031548		
	chr5	36200001:36300000	20	rs137189970	rs134607051	0.011137951	*TMEM117*	Bos taurus transmembrane protein 117
	chr9	47500001:47600000	19	rs137052587	rs137140432	0.024563868		

### Saturated fatty acids

**Fig. 2:**
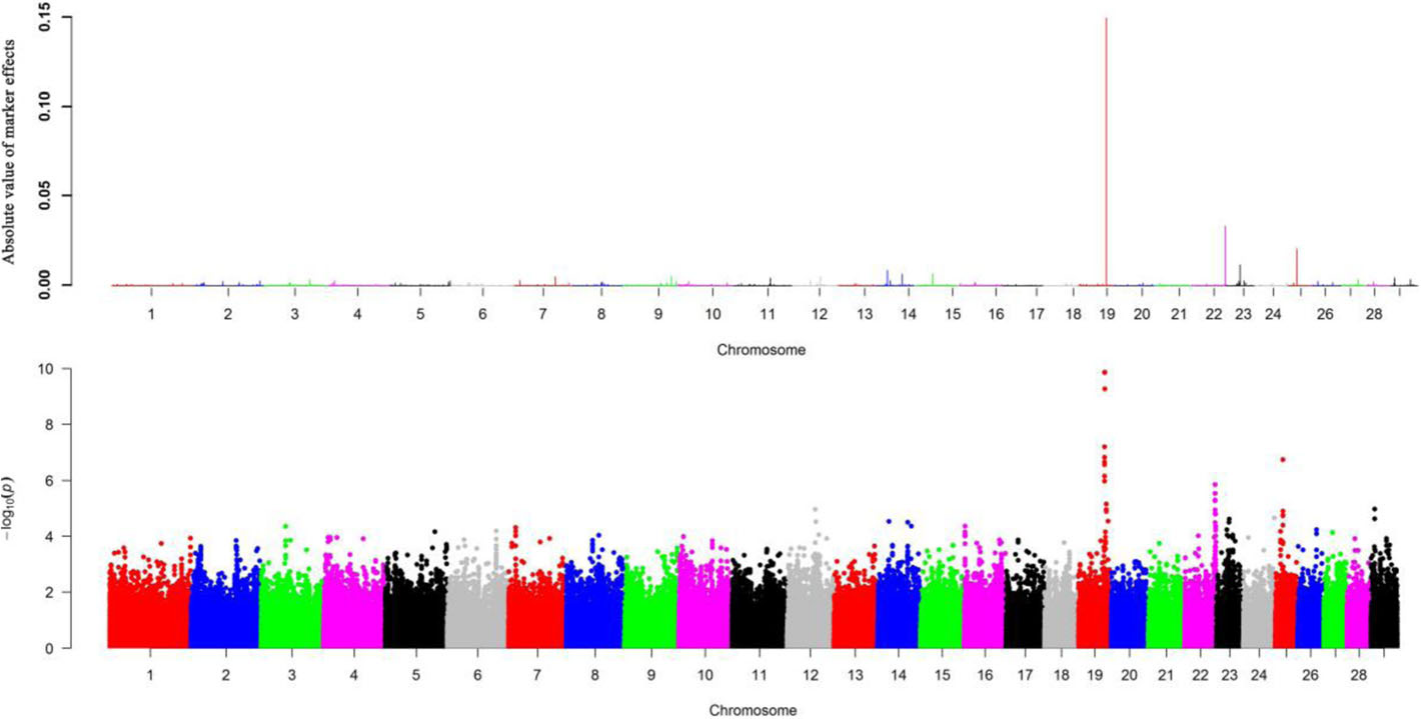
**a** Manhattan plot of absolute value of SNP effects estimated using BayesB for C14:0. **b** Manhattan plots showing *P*-values of association for each SNP using the GRAMMAR-GC, where the y-axis was defined as -Log 10 (*P*)

We detected a total of 16 candidate regions that explain more than 1% of genetic variance for saturated fatty acids. These regions were distributed on BTA2, BTA4, BTA6, BTA7, BTA12, BTA14, BTA15, BTA19, BTA22, BTA23 and BTA25 (Table 2). Among them, we found four, three and nine candidate regions for C14:0, C18:0 and C20:0, respectively (Additional file 2). Intriguingly, the detected window with the largest genetic variance (10.04%) near 51.3 Mb on BTA19 for C14:0, containing gene fatty acid synthase (*FASN*) that is related to fatty acid synthesis. We also found one region explaining about 1.46% of genetic variance for C14:0 and located at 25.1 Mb on BTA23. This region overlapped with the elongation of very long chain fatty acids protein 5 (*ELOVL5*) whose function involves in fatty acid elongase activity (Fig. 2). In addition, we identified 10 candidate genes that are likely to be related to fatty acids composition embedded in these candidate regions (Table 2).

### Monounsaturated fatty acids

**Fig. 3:**
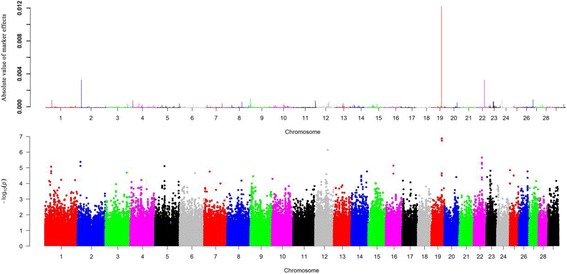
**a** Manhattan plot of absolute values of SNP effects estimated using BayesB for C14:1 cis-9. **b** Manhattan plots showing *P*-values of association for each SNP using the GRAMMAR-GC, where the y-axis was defined as -Log 10 (*P*)

**Fig. 4:**
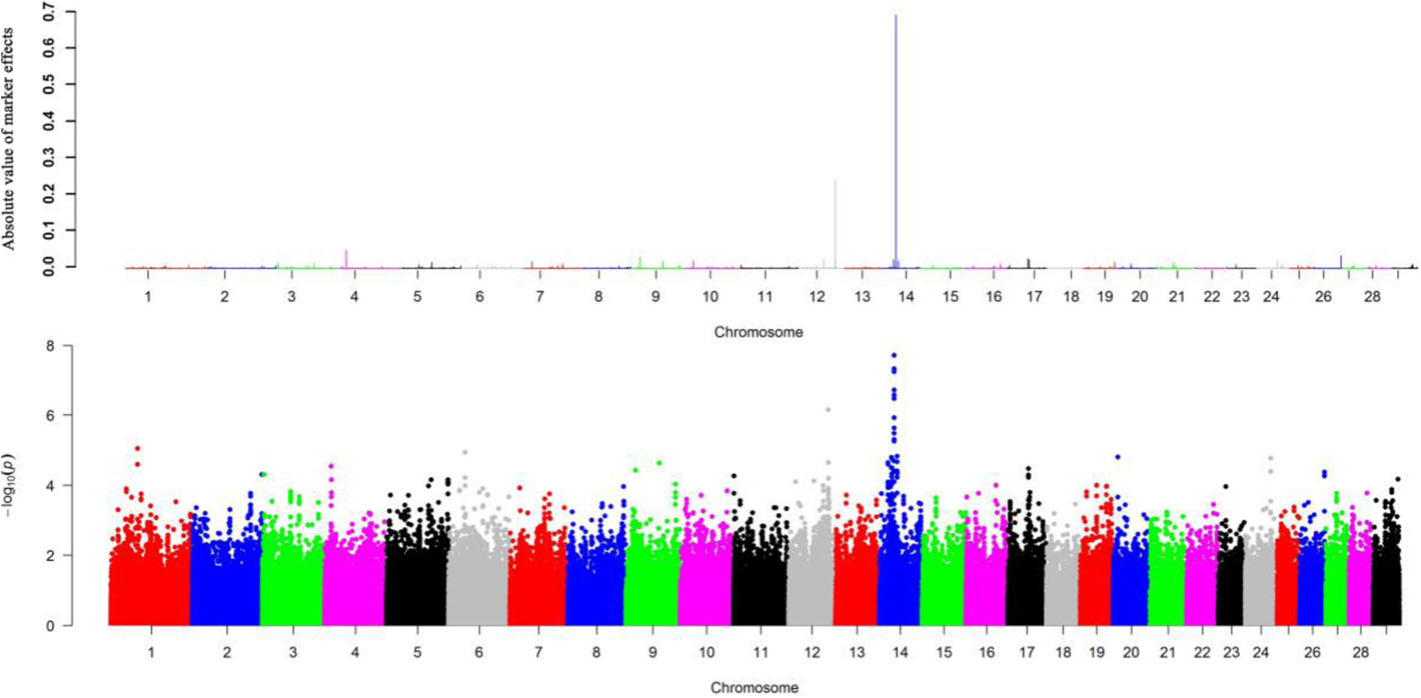
**a** Manhattan plot of absolute values of SNP effects estimated using BayesB for C18:1 cis-9. **b** Manhattan plots showing *P*-values of association for each SNP using GRAMMAR-GC, where the y-axis was defined as -Log 10 (*P*)

For monounsaturated fatty acids, we detected 8 candidate regions, each of which captured more than 1% of the total genetic variance across six chromosomes (Additional file 3). Notably, we detected the same region at 51.3 Mb on BTA19 overlapping with *FASN*, which explains 6.49% of the genetic variance for C14:1 cis-9 (Fig. 3), and this region was also associated with C14:0. The top associated region was detected near 30.3 Mb on BTA14, which explains 19.44% of genetic variance for C18:1 cis-9 (Fig. 4), while no known gene was observed near this region. Overall, there are three, two and three regions identified to be associated with C14:1 cis-9, C16:1 cis-9 and C18:1 cis-9, respectively (Table 3).

### Polyunsaturated fatty acids

We detected 11 associated regions for five polyunsaturated fatty acids, including C18:2 n-6, C18:3 n-3, C20:3 n-3, C20:5 n-3 and C22:5 n-3 (Additional file 4). Of these regions, we observed one candidate region for C18:3 n-3 that was located at BTA14, two regions associated with C18:2 n-6 at BTA4 and BTA20, two regions associated with C20:3 n-3 at BTA4 and BTA17, three regions associated with C20:5 n-3 at BTA3, 5, 12, and three regions associated with C22:5 n-3 at BTA2, 5, 9 respectively (Table 3). Notably, we found 12 genes imbedded in the identified regions and these genes were likely involved in the function of fatty acid synthesis and metabolism. Of these 12 genes, two genes were associated with C18:2 n-6, three genes with C20:3 n-3, six genes with C20:5 n-3, and one gene with C22:5 n-3, respectively. The top four regions explaining more than 2% of the genetic variance were identified at BTA20 associated with C18:2 n-6 (4.95%), BTA14 with C18:3 n-3 (2.83%), BTA9 with C22:5 n-3 (2.46%) and BTA4 with C18:2 n-6 (2.33%).

### Fatty acid groups

To systematically explore the genetic mechanism underlying fatty acid composition beyond individual fatty acid, we also conducted GWAS using BayesB method on eight fatty acid groups, including SFA, MUFA, PUFA, PUFA/SFA, n-3, n-6, n-6/n-3, and HI (Additional file 5). We found three associated regions for HI located at BTA4, BTA10 and BTA15, two regions for MUFA at BTA12 and BTA14, one for n-3 at BTA4 and one for n-6/n-3 at BTA20 (Table 4). Intriguingly, we observed two regions accounting for ~10% of the genetic variance for MUFA, while two regions located at BTA4 and BTA20 accounting for 1.25% and 4.66% of genetic variance for n-3 and n-6/n-3, respectively. One region located at BTA12 overlapped with two genes claudin 10 (*CLDN10*) and DAZ interacting zinc finger protein 1 (*DZIP1*). For health index (HI), the genetic variances contributed by three identified regions were 1.52%, 1.19% and 2.03%, which located at BTA4, BTA10 and BTA15, respectively. These regions overlapped with sarcoglycan epsilon (*SGCE*), paternally expressed 10 (*PEG10*) and DEAD-box helicase 10 (*DDX10*).

**Table 4:** Genomic regions associated with the fatty acid groups in Chinese Simmental cattle using BayesB method

FA Trait	Chr	Associated Window	nSNP	rs_ID_start	rs_ID_end	Genetic variance for 100 kb window	Positional/putative candidate gene	Gene name
MUFA	chr14	30300001:30400000	27	rs135047094	rs41733749	0.09885106		
	chr12	76800001:76900000	43	rs42705033	rs133235260	0.104124564	*CLDN10* *DZIP1*	Bos taurus claudin 10 Bos taurus DAZ interacting zinc finger protein 1
n-3	chr4	89200001:89300000	28	rs110084128	rs133410381	0.012452369		
n-6/n-3	chr20	22500001:22600000	26	rs110568039	rs137611969	0.046649777		
HI	chr15	18900001:19000000	34	rs134293788	rs110280875	0.020338987	*DDX10*	Bos taurus DEAD-box helicase 10
	chr4	11900001:12000000	26	rs137053488	rs110825076	0.015207173	*SGCE* *PEG10*	Bos taurus sarcoglycan epsilon Bos taurus paternally expressed 10
	chr10	8200001:8300000	28	rs136862543	rs137767957	0.011854547		

### Identification of associated loci using GRAMMAR-GC

We further conducted GWAS using GRAMMAR-GC implemented in GenABEL package for 11 individual fatty acids and eight fatty acid groups, each of which has a genomic heritability of high than 0.10. To ensure the power and accuracy of GWAS for these traits, we utilized genomic control approach to correct for possible population stratifications in GRAMMAR-GC test. After this correction, we found the inflation factor λ was close to one, suggesting that our approach has successfully accounted for population stratification, and thus no further adjustment was required. We identified a total of 44 and 8 significant SNPs associated with nine fatty acid composition and two fatty acid groups, respectively. The suggestive *P* value (0.05/163,473 = 3.06E-7) was used as the cut off threshold for significance, which approximately considered the number of “independent” SNPs by counting 1 SNP per LD block, plus all SNPs outside of blocks (interblock SNPs). We observed 14, 5, 8, 1, 3, 4, 3, 3 and 3 significant associated SNPs surpassing the suggestive threshold (*P* <3.06E-7) for C14:0, C14:1 cis-9, C18:1 cis-9, C18:3 n-6, C20:0, C20:1 cis-11, C20:2 n-6, C20:4 n-6 and C18:2 t-9c-11, respectively. The top four significant SNPs for C14:0 (*P* =1.39E-10) were located at 51.3 Mb on BTA19. Totally, we identified 17 associated SNPs for saturated fatty acid (C14:0 and C20:0), 17 SNPs for monounsaturated fatty acids (C14:1 cis-9, C18:1 cis-9 and C20:1 cis-11), 10 SNPs for polyunsaturated fatty acids (C18:3 n-6, C20:2 n-6, C20:4 n-6 and C18:2 t-9c-11). Notably, we found the majority of SNPs were located at BTA19 (18 SNPs) and BTA14 (19 SNPs), which indicated these regions are potential candidate for fatty acid composition. Fig. 2, 3 and 4 show the genome-wide plots of C14:0, C14:1 cis-9 and C18:1 cis-9 for *P*-values and the absolute values of marker effects against the genomic position. We found one associated SNP located at 51.3 Mb on BTA19 for both C14:0 and C14:1 cis-9 (*P* = 5.19E-10 and *P* = 1.82E-07), and this SNP was also located at ~4 kb upstream of the *FASN* gene.

### Region-based association test and LD analyses

**Fig. 5:**
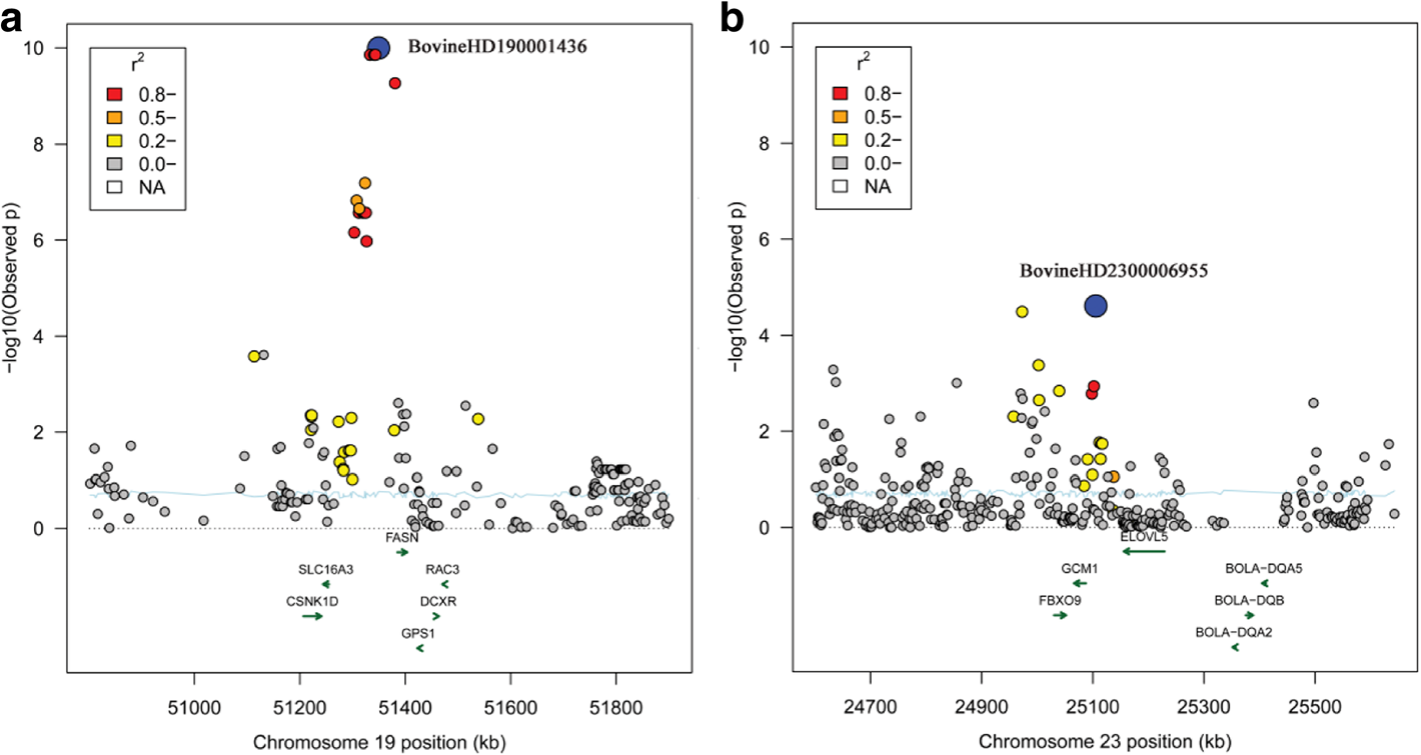
Regional plots of the two major candidate regions on BTA19 and BTA23. Results were shown for C14:0 at 50.8-51.8 Mb on BTA19 (**a**) and for C20:0 around 24.6-25.6 Mb on BTA23 (**b**). In the upper panels, the top SNPs were highlighted by blue solid circles. Different levels of linkage disequilibrium (LD) between the lead SNP and surrounding SNPs were indicated in different colors

To explore potential associated loci which might fail to be identified due to the strict threshold for high density SNPs, we investigated two 100 kb associated regions on BTA19 and BTA23 (BTA19:51.3–51.4 Mb and BTA23: 25.1–25.2 Mb) using region-based association tests implemented in R package FREGAT. The two regions contain two candidate genes *FASN* and *EVOL5* involved in fatty acid synthesis. For the region at 51.3 Mb on BTA19, we found 19 SNPs showing significant association with C14:0 (*P* < 0.01), and among them, five SNPs were identified within *FASN*, and one SNP near *FASN* with the strongest association signal (*P* = 5.17E-10). We found that the *P* value for region-based test for *FASN* was 0.0048, which indicated that *FASN* may be considered as a candidate gene for C14:0. Moreover, the LD and haploblock analyses revealed that this region showed high LD level with multiple haploblocks, which may imply a potential selection signature involved in fatty acids within this region in Chinese Simmental cattle population (Fig. 5a). For region at 25.1 Mb on BTA23, two SNPs were detected with *P* < 0.01, and the top SNP (BovineHD2300006955) was detected at 25.1 Mb showing significant association (*P* = 3.1E-5). This region partly overlapped with gene *ELVOL5*. Therefore, we next examined the 500 kb upstream and downstream of the region. However, no other SNPs were found which were significantly associated with C14:0 (Fig. 5b).

### Genomic prediction for fatty acid composition

We performed genomic selection for fatty acid composition using GBLUP and BayesB. The predictive accuracies ranged from 0.03 (C18:2 t-12c-10) to 0.51 (C18:3 n-3) using GBLUP, and from 0.1 (C18:2 t-9c-11) to 0.53 (C14:0) using BayesB. The averaged predictive accuracies across all fatty acids using GBLUP and BayesB were 0.24 and 0.29, respectively. These results suggested that genomic prediction using BayesB was slightly superior over GBLUP for fatty acids in Chinese Simmental cattle.

For each individual fatty acid trait, we found that the relatively high predictive accuracies were achieved for C14:0, C22:0, C14:1 cis-9, C18:3 n-3, C20:3 n-3 for both GBLUP and BayesB. Despite the fact that BayesB performed at least as well as GBLUP for most traits, we indeed found some traits where BayesB showed much higher predictive accuracies than GBLUP, such as C14:0 (0.48 for GBLUP and 0.53 for BayesB), C18:0 (0.17 vs. 0.24), C24:0 (0.11 vs. 0.23), C18:2 t-12c-10 (0.03 vs. 0.12), C18:3 n-6 (0.05 vs. 0.13), C20:2 n-6 (0.07 vs. 0.14) (Table 5). To investigate possible bias between the predicated and observed breeding values, we also calculated the regression coefficient of genomic estimated breeding values on adjusted phenotypes (Table 5). Our results revealed that the average regression coefficients for GBLUP and BayesB were 0.71 and 0.93, indicating that BayesB is less biased than GBLUP because the latter has a regression coefficient closer to unity.

**Table 5:** Predictive accuracy (±SE) and regression coefficients (±SE) of genomic breeding value prediction for fatty acid composition

Trait	GBLUP		BayesB	
	r	b	r	b
C14:0	0.48 ± 0.08	0.58 ± 0.05	0.53 ± 0.06	0.94 ± 0.08
C16:0	0.36 ± 0.06	0.72 ± 0.13	0.39 ± 0.1	1.08 ± 0.21
C18:0	0.17 ± 0.09	0.68 ± 0.15	0.24 ± 0.09	0.78 ± 0.19
C20:0	0.39 ± 0.1	0.62 ± 0.18	0.42 ± 0.06	1.09 ± 0.18
C22:0	0.45 ± 0.05	0.74 ± 0.11	0.48 ± 0.07	1.21 ± 0.28
C24:0	0.11 ± 0.07	0.89 ± 0.13	0.23 ± 0.08	0.63 ± 0.14
C14:1 cis-9	0.41 ± 0.06	0.81 ± 0.09	0.45 ± 0.08	1.02 ± 0.12
C16:1 cis-9	0.16 ± 0.04	0.63 ± 0.22	0.2 ± 0.04	0.69 ± 0.23
C18:1 cis-9	0.25 ± 0.08	0.72 ± 0.24	0.28 ± 0.06	1.25 ± 0.41
C20:1 cis-11	0.16 ± 0.06	0.88 ± 0.15	0.18 ± 0.06	1.26 ± 0.24
C18:2 n-6	0.15 ± 0.07	0.87 ± 0.19	0.18 ± 0.07	1.16 ± 0.15
C18:2 t-9c-11	0.04 ± 0.08	1.07 ± 0.12	0.1 ± 0.06	0.71 ± 0.34
C18:2 t-12c-10	0.03 ± 0.07	1.08 ± 0.14	0.12 ± 0.05	0.82 ± 0.32
C18:3 n-6	0.05 ± 0.08	0.33 ± 0.25	0.13 ± 0.06	0.92 ± 0.12
C18:3 n-3	0.51 ± 0.06	1.08 ± 0.17	0.53 ± 0.08	1.18 ± 0.21
C20:2 n-6	0.07 ± 0.05	0.12 ± 0.11	0.14 ± 0.05	0.06 ± 0.21
C20:3 n-3	0.42 ± 0.06	0.76 ± 0.28	0.47 ± 0.08	1.54 ± 0.62
C20:4 n-6	0.26 ± 0.09	0.56 ± 0.17	0.31 ± 0.06	0.65 ± 0.32
C20:5 n-3	0.16 ± 0.06	0. 40 ± 0.32	0.2 ± 0.09	0.96 ± 0.41
C22:5 n-3	0.22 ± 0.05	0.45 ± 0.28	0.25 ± 0.07	0.83 ± 0.36
C22:6 n-3	0.24 ± 0.06	0.51 ± 0.24	0.25 ± 0.05	0.89 ± 0.29

## Discussion

Fatty acid is an important indicator of meat quality and taste, and its strongly influences consumer’s preferences [3, 38]. Previous genome-wide association studies have been conducted for fatty acid composition in multiple cattle breeds, including Angus, Japanese Black, Nellore and other crossbreds [8, 30, 32, 35, 36]. To our knowledge, this study is the first attempt to investigate molecular mechanism underling fatty acid composition using high density SNP array and evaluate the accuracy of genomic predictions in Chinese Simmental cattle population.

### Genomic wide scan identified candidate regions and loci

We investigated 21 individual fatty acids including 6 saturated fatty acids, 4 monounsaturated fatty acids, 11 polyunsaturated fatty acids and 8 fatty acid groups. Despite the fact that single-SNP based GWAS methods have been widely used in many studies, these methods may not be powerful for studying the complex traits with low or moderate heritability. Due to the polygenic characteristics of fatty acid composition in cattle, GWAS using the Bayesian methods have enabled to identify many associated loci that have missed by the single-SNP regression approach [33, 35, 36]. BayesB has been widely used for GWAS of complex trait in farm animals [39–42]. In this study, we utilized BayesB and GRAMMAR-GC to identify candidate regions associated with fatty acids in Chinese Simmental cattle population. We detected a total of 35 candidate regions on 16 autosomes associated with fatty acid composition using BayesB. However, these identified regions may not include all potentially significant associated SNPs due to use of 100 kb window-based strategy in BayesB. Therefore, we conducted GWAS using the single locus GRAMMAR-GC method implemented in GenABLE package. With this approach, we detected a total of 44 and 8 significant associated SNPs for individual fatty acids and fatty acid groups using a suggestive adjust threshold. The suggested threshold was set to avoid overestimation of the significant SNPs caused by high LD level in the high density SNPs array [43]. In current study, we found a total of 24 candidate SNPs overlapping with these regions identified by BayesB. For instance, the same candidate peaks for C14:1, C14:1 cis-9 and C18:1 cis-9 were identified using both methods (Figs. 2–4). Utilization of multiple complementary methods is an effective way to detect candidate regions or SNPs and helps elucidate genetic architecture of complex traits in farm animals [33, 44].

### Candidate genes for fatty acid composition

Several genes were identified as potential candidates contributing to the genetic architecture of fatty acids in this study. Among them, we observed *FASN* at 51 Mb on BTA19 overlapped with a 100 kb associated region, which explains 10% and 6.5% of the genetic variances for C14:0 and C14:1 cis-9, respectively. Notably, we also found multiple significant SNPs around this gene that were associated with C14:0 using the GRAMMAR-GC method. A region-based association test revealed strong evidence of association for multiple SNPs within this gene. Furthermore, we found strong LD at the upstream of *FASN* (Fig. 5). This gene encodes a multifunctional protein enzyme to catalyze the synthesis of palmitate (C16:0) from acetyl-CoA and malonyl-CoA, in the presence of *NADPH*. Previous studies based on candidate gene approaches had revealed polymorphisms within *FASN* that were related to fatty acid composition in multiple beef cattle populations [45–49] and milk fat content in dairy cattle [50, 51]. For instance, several studies were conducted to explore and evaluate the association between fatty acid composition and candidate SNPs using GWAS in Japanese beef cattle [8, 30, 52]. These studies provided multiple evidences that several SNPs near or within *FASN* may be regarded as responsible mutations for fatty acid composition and contribute largely to meat quality in the Japanese Black cattle population. Saatchi *et al* performed GWAS using BovineSNP50 in Angus beef cattle and reported *FASN* located at 51 Mb on BTA19 (from 51,384,922 to 51,403,614 bp) was associated with fatty acids [32]. Chen et al. found SNP rs41921177 (BTA19:51,326,750) located near *FASN*. This SNP rs41921177 had relative large effects on multiple fatty acids in both subcutaneous adipose and *longissimus lumborum* muscle tissues of crossbred beef cattle [33]. However, investigation of genetic architecture of fatty acids in the Nellore cattle showed no significant associations for several polymorphisms within or near *FASN* [35, 36]. Despite previous studies have indicated that *FASN* has a conserved role across genetic backgrounds, there are several different variants that may be responsible for the different *FASN* effects in different breeds, and different *FASN* alleles appear to be segregating in different populations [8, 49].

Another gene called *ELOVL5* encodes a multi-pass membrane protein which is involved in the elongation of long-chain polyunsaturated fatty acids. This gene was identified in the associated region at 25.1 Mb on BTA23 accounting for 1.5% of the genetic variance for C14:1 cis-9. *ELOVL5* plays an important role in *de novo* synthesis of specific MUFA species in mammalian cells, *ELOVL5* knockdown decreased the elongation of C16:1 cis-9, n-7, and *ELOVL5* overexpression increased synthesis of C18:1 cis-9, n-7 [53]. Also, previous study using mice models revealed that a reduced *ELOVL5* activity can lead to hepatic steatosis, and endogenously synthesized PUFAs are critical regulators of fatty acid synthesis [54]. Lemos et al. reported a candidate region embedded in *ELOVL5* can explain 4% of the genetic variance for C20:4 n-6 using ssGBLUP based on window association test in Nellore cattle [35]. The consistent role of *ELOVL5* gene involved in fatty acid synthesis and composition was also extensively investigated in diverse pig populations [55–57].

Moreover, previous studies suggested that *ELOVL5* are involved in the production of multiple acids including C16:0, C16:1, C18:0 and C18:1 in cattle [58]. Also, *ELOVL5* was found associated with C20:1n9/C18:1n9 and C20:2n6/C18:2n6 in a F2 population derived from Erhulian pig [55]. As a result, *ELOVL5* may have pleiotropic effects on multiple fatty acid composition and also appear to exhibit pleiotropic effects in multiple metabolic steps. However, we only identified *ELOVL5* that was associated with C14:0 in Chinese Simmental cattle. In addition, several previous studies have suggested variants within *SCD* gene and the expression level of *SCD* gene should be significantly associated with fatty acids [18, 20, 30, 32, 47, 48, 59, 60], the *SCD* gene was not detected in this study, probably due to heterogeneous genetic architecture of fatty acids differ across different populations.

### Genomic prediction for fatty acid composition

Previous studies have investigated predictive abilities of genomic selection for fatty acid composition in American Angus [32], Japanese Black cattle [61] and Canadian beef cattle [33]. In the current study, we explored, for the first time, genomic prediction for fatty acid composition in Chinese Simmental cattle. We found that the accuracies of genomic prediction for most of fatty acids were relatively low (<0.30) using both GBLUP and BayesB, which was consistent with a previous report by Chen et al. [33]. This finding may be explained by the relatively low and inaccurate estimates of heritability for the measured fatty acid composition [62]. Our studies also revealed that BayesB provided slightly higher average regression coefficients as compared to GBLUP. Considering the complex architecture of fatty acid composition, this finding implied that BayesB which allows a fraction of SNPs to be allocated with relatively large effects is superior over GBLUP which assumes the same genetic variance for each SNP. Fatty acid composition are commonly recognized as complex traits with a polygenic nature and, to some extent, they are difficult to measure, thus the application of genomic selection for fatty acids will be valuable in future selection breeding programs. With increasing public understanding of the relationships between diet and health, much attention should be paid to the studies of some important fatty acids related to human health [63]. As consumer become more health conscious, they have increased preference for better tasting and healthier products in their diet such as unsaturated fatty acid levels. Further investigation of causal mutations will promote our understanding of lipid metabolism, fat deposition and application of selection for fatty acids in cattle.

## Conclusions

We identified several significant associated regions and loci as the potential candidate markers for genomics-assisted breeding programs. Using multiple methods, our results revealed that *FASN* and *ELOVL5* associated with fatty acids with strong evidences. Our analyses also suggested that it is feasible to perform genomic selection for fatty acids in the Chinese Simmental cattle population.

## Methods

### Ethics statement

All animals used in the study were treated following the guidelines established by the Council of China Animal Welfare. Protocols of the experiments were approved by the Science Research Department of the Institute of Animal Sciences, Chinese Academy of Agricultural Sciences (CAAS) (Beijing, China).

### Animals and phenotypes

A total of 723cattleborn between 2010 and 2013 were used in this study, and these cattle were originated from Ulgai, Xilingol League and Inner Mongolia of China. After weaning, the cattle were moved to JinweifurenCo.,Ltd for fattening, all animals sharing the same feeding and management conditions. More detailed description of breeding and management has been described previously [64, 65]. The cattle were slaughtered at an average of 20 months. During the period of slaughtering, we measured traits in strict accordance with the guidelines proposed by the Institutional Meat Purchase Specifications for fresh beef. Meat samples were removed from the *longissimus lumborum* (LL) muscleafter stored for 48 h between the 12th and 13th ribsfrom each animal, and then samples were vacuum packed and chilled at -80 °C. And approximate 10 g of sample were taken for subsequent fatty acid analyses. Total lipid was extracted from the sample according to protocols described previously [66]. About 2 mg extracted lipid was re-dissolved in 2 ml of n-hexane and 1 ml of KOH (0.4 M) for saponification and methylation. A total of 21 individual fatty acid composition were measured using gas chromatography (GC-2014 CAFsc, Shimadzu Scientific Instruments) including six saturated fatty acids, four monousaturated fatty acids, and eleven polyunsaturated fatty acids. Each fatty acid was quantified as a weight of percentage of total fatty acids. In addition, fatty acids were indexed as groups of saturated, monounsaturated, polyunsaturated fatty acid, total of saturated fatty acid (SFA), total monounsaturated (MUFA), total of polyunsaturated (PUFA), total of omega 3 (n-3) and total of omega 6 (n-6). The calculation of various fatty acid groups are described as follows: SFA = C14:0 + C16:0 + C18:0 + C20:0 + C22:0 + C24:0; MUFA = C14:1 cis-9 + C16:1 cis-9 + C18:1 cis-9 + C20:1 cis-11; PUFA = C18:2 n-6 + C18:2 t-9c-11 + C18:2 t-12c-10 + C18:3 n-6 + C18:3 n-3+ C20:2 n-6 + C20:3 n-3 + C20:4 n-6 + C20:5 n-3 + C22:5 n-3 + C22:6 n-3; n-3 = C18:3 n-3 + C20:3 n-3 + C20:5 n-3 + C22:5 n-3 + C22:6 n-3; n-6 = C18:2 n-6 + C18:3 n-6 + C20:2 n-6 + C20:4 n-6; PUFA/SFA: ratio between PUFA and SFA; n-6/n-3: ratio between n-6 and n-3; HI = (MUFA + PUFA)/(4 × C14:0 + C16:0).

### Genotyping and quality control

In total, 723 Simmental cattle were genotyped for the Illumina BovineHD BeadChip. Before statistical analysis, SNPs were pre-processed using PLINK v1.07 [67]. SNPs were selected based on minor allele frequency (>0.05), proportion of missing genotypes (<0.05), and Hardy-Weinberg equilibrium (*P* > 10E-6). Moreover, individuals with more than 10% missing genotypes were excluded. After these quality controls, the final data consisted of 685 individuals and 595,715 autosomal SNPs.

### Heritability and genetic correlation estimation

Phenotypic and genetic (co) variances of fatty acids were estimated using the pairwise bivariate animal model implemented in the ASReml v3.0 software package [68]. The model is

$$ \left[\begin{array}{c}\hfill {y}_1\hfill \\ {}\hfill {y}_2\hfill \end{array}\right]\kern0.5em =\kern0.5em \left[\begin{array}{cc}\hfill {X}_1\hfill & \hfill 0\hfill \\ {}\hfill 0\hfill & \hfill {X}_2\hfill \end{array}\right]\left[\begin{array}{c}\hfill {b}_1\hfill \\ {}\hfill {b}_2\hfill \end{array}\right]+\kern0.5em \left[\begin{array}{cc}\hfill {Z}_1\hfill & \hfill 0\hfill \\ {}\hfill 0\hfill & \hfill {Z}_2\hfill \end{array}\right]\left[\begin{array}{c}\hfill {a}_1\hfill \\ {}\hfill {a}_2\hfill \end{array}\right]\kern0.5em +\left[\begin{array}{c}\hfill {\mathbf{e}}_{\mathbf{1}}\hfill \\ {}\hfill {\mathbf{e}}_{\mathbf{2}}\hfill \end{array}\right] $$

where y_1_ and y_2_ are vectors of phenotypic values of trait 1 and 2, respectively; ×_1_ and *×*_2_ are incidence matrices for fixed effects; *b*_*1*_ and *b*_*2*_ are the vectors of the fixed effects; Z_1_ and Z_2_ are incidence matrices relating the phenotypic observations to vectors of the polygenic (*a*) effects for two traits; **e**_**1**_ and **e**_**2**_ are random residuals for two traits. The polygenic effects were treated as random and assumed to be mutually independent.

Variances of the random effects are defined as *V*(*a*) = **G** *σ*_*a*_^2^ for the polygenes and *V*(*e*) = **I** *σ*_*e*_^2^ for the residuals, where G is the additive genetic relationship matrix, **I** is the identity matrix, *σ*_*a*_^2^ is the additive genetic variance and *σ*_*e*_^2^ is the residual variance. Matrix G matrix was inferred from the SNP markers according to the study of VanRaden [69]. Fixed effects in the model included effects of gender, farm and year. In addition, ages at slaughter, days between slaughter and fatty acid extraction, hot carcass weight and marbling score were considered as covariates in the model. Genomic heritability of each trait was estimated using

$$ {h}^2={\sigma}_a^2/\left({\sigma}_a^2,+,{\sigma}_e^2\right) $$

Pairwise bivariate analyses were performed for each combination of fatty acids to estimate the (co) variance components, phenotypic and genetic correlations as well as the heritability.

### Genome-wide association study using BayesB

Fatty acid composition was adjusted for fixed effects and covariates using a linear mixed model, and fixed effects and covariates were defined above. We conducted genome-wide association analyses using BayesB, which analyzed all autosomal SNP simultaneously and assumed different genetic variance for each SNP [40, 70]. The model is described as follows,

1 $$ {y}_i= u+{\displaystyle \sum_{j=1}^M{Z}_{i j}{\alpha}_j{\delta}_j+{e}_i} $$

where *y*_*i*_ is the adjusted phenotypic value for the *i* th individual, *u* is the mean (after removing fixed effects and all covariants), M is the number of SNP loci, *Z*_*ij*_ is the *j* th SNP genotype of animal *i* coded as the number of B alleles in the genotype, *α*_*j*_ is the average effect of allele substitution for SNP *j*, and is assumed to be normally distributed N (0, *σ*_*j*_^2^), *δ*_*j*_ is an indicator variable to show the presence (*δ*_*j*_ = 1) and absence (*δ*_*j*_ = 0) of marker *j*, and the presence is given a prior probability, and *e*_*i*_ is the residual error with an assumed normal distribution N (0, *σ*_*e*_^2^). The prior distribution of variance *σ*_*j*_^2^ (or *σ*_*e*_^2^) is assumed to be a scaled inverse Chi-square with degrees of freedom *v*_*α*_ = 4 (or *v*_*e*_ = 10) and scale parameter *S*_*α*_^2^ (or *S*_*e*_^2^). The scale parameter was usually derived from an assumed additive-genetic variance [71]. *π* was set to 0.9998, which meant that about 100–150 SNPs were fitted simultaneously in each MCMC iteration. The Markov chains were run for 50,000 cycles of iterations with the first 10,000 iterations being discarded as burn-in followed by additional 40,000 iterations to form the posterior sample. All SNPs effects were estimated from the posterior sample. We performed GWAS for all the 21 fatty acids but only reported the results for the traits with genomic heritability ≥ 0.10. We inferred the associations for fatty acids using a 100 kb window rather than single marker [35, 36]. There were 24,900 SNP windows across the 29 autosomes. The variance for each window was estimated using the genetic value of all adjacent SNPs within 100 kb window, and proportion of genetic variance explained by each window was obtained by dividing the variance of window breeding value by the variance of the whole genome breeding value. Genome windows with the highest posterior mean proportion of genetic variance ≥1% were considered as the most important regions associated with the traits. Positional candidate genes were investigated for the 100 kb windows using the UCSC Genome Browser, which allowed visualization of SNP based on the *Bos taurus* genome assembly UMD 3.1.

### Genome wide association study using GRAMMAR

We also performed genome-wide association study using GRAMMAR-GC implemented in an R package GenABEL v1.8-0 [72]. The method accounts for population stratification and covariance structure of individuals inferred from all by SNPs. Bonferroni corrected threshold of 8.39E-08 (0.05/595,715) was adopted for the top 5% genome-wide significance. This correction was highly conservative for GWAS using high density SNPs array. To avoid the “overcorrection” for SNPs that may not truly independent due to LD across genome, we used a suggestive *P* value (*P* = 0.05/163,473) as thresholds proposed by Duggal et al., considering approximate the number of “independent” SNPs by counting 1 SNP per LD block, plus all SNPs outside of the LD blocks (interblock SNPs) [43].

### Region-based association test and haploblock analyses

Region-based association test is a more powerful approach of gene mapping than the association test of an individual genetic variant. In this study, we performed the region-based association test for several target 100 kb regions identified using BayesB. SNPs in these regions and the adjusted fatty acid composition were investigated with this region-based association test using R package FREGAT [73]. The LD of these regions were estimated using PLINK v1.7 software [67].

### Genomic prediction

Genomic best linear unbiased prediction (GBLUP) and BayesB were used for genomic prediction. Five-fold cross validation was used to evaluate the accuracy of genomic prediction. The data were split into five approximately equal-sized groups. For each cross validation, four groups were used as the training sample to estimate parameters and the remaining group was used as the test sample. The linear model is written as,

2 $$ \mathbf{y}={\mathbf{1}}_{\mathbf{n}} u+\mathbf{Z}\mathbf{a}+\mathbf{e} $$

where y is the vector of adjusted phenotypic values in the training sample, *μ* is the overall mean, **a** is the vector of breeding values for all animals, **e** is the vector of residuals errors and **Z** is the incidence matrix for the random effects. For the BayesB, SNP effects were estimated based on the training population using the statistical model described in the GWAS analyses. The GEBV for animal *i* in the validation population was predicted by summing up SNP effects over all loci as follows: *GEBV*_*i*_ = ∑_*j* = 1_^*M*^ *Z*_*ij*_ *α*_*j*_, where *α*_*j*_ is the estimated effect for SNP *j*. Predictive accuracy was measured as the correlation between the estimated breeding values and the adjusted phenotypic values divided by the square root of heritability separately for each of the 5-fold cross-validation replicates.

## Additional files

Additional file 1: Table S1. Estimates of phenotypic correlations (upper diagonals) and genetic correlation (lower diagonals) between 21 phenotypes in Chinese Simmental beef cattle. (DOCX 19 kb)

Additional file 2: Summary statistics of the 100 kb regions for 3 saturated fatty acids. Results include trait name, chromosome, 100 kb windows on the UMD3.1 genome assembly, window variances, and percentage of genetic variance using BayesB. (CSV 2102 kb)

Additional file 3: Summary statistics of the100 kb regions for three monounsaturated fatty acids (MUFA). Results include trait name, chromosome, 100 kb windows on the UMD3.1 genome assembly, window variances, and percentage of total genetic variance using BayesB. (CSV 2074 kb)

Additional file 4: Summary statistics of the 100 kb regions for five polyunsaturated fatty acids (PUFA). Results include trait name, chromosome, 100 kb windows on the UMD3.1 genome assembly, window variances, and percentage of genetic variance using BayesB. (CSV 2967 kb)

Additional file 5: Summary statistics of 100 kb regions for four fatty acid groups. Results include trait name, chromosome, 100 kb windows on the UMD3.1 genome assembly, window variances, and percentage of genetic variance using BayesB. (CSV 2520 kb)

## Abbreviations

BTA: Bos taurus chromosome
GBLUP: Genomic best linear unbiased prediction
GWAS: Genome-wide association study
HI: Health index
MUFA: Sum of monounsaturated fatty acids
n-3: Sum of omega 3 acids
n-6: Total of omega 6 acids
n-6/n-3: Ratio between n-6 and n-3 PUFA
PUFA: Sum of polyunsaturated fatty acid
QTL: Quantitative trait loci
SE: Standard error
SFA: Sum of saturated fatty acids
SNP: Single nucleotide polymorphism
