# Supplementary material for: Genome wide association study and genomic prediction for fatty acid composition in Chinese Simmental beef cattle using high density SNP array
Source: BMC Genomics. 2017 Jun 14;18:464. doi: 10.1186/s12864-017-3847-7 (PMC5471809; doi:10.1186/s12864-017-3847-7)
Supplement: Supplementary file 1 — Estimates of phenotypic correlations (upper diagonals) and genetic correlation (lower diagonals) between 21 phenotypes in Chinese Simmental beef cattle. (DOCX 19 kb) [file 12864_2017_3847_MOESM1_ESM.docx]

Additional Table 1. Estimates of phenotypic correlations (above diagonal) and genetic correlation (below diagonal) between 21 phenotypes in Simmental beef cattle.

| Trait^a^ | C14:0 | C16:0 | C18:0 | C20:0 | C22:0 | C24:0 | C14:1 cis-9 | C16:1 cis-9 | C18:1 cis-9 | C20:1 cis-11 | C18:2 n-6 | C18:2 t-9c-11 | C18:2 t-12c-10 | C18:3 n-6 | C18:3 n-3 | C20:2 n-6 | C20:3 n-3 | C20:4 n-6 | C20:5 n-3 | C22:5 n-3 | C22:6 n-3 |
| --- | --- | --- | --- | --- | --- | --- | --- | --- | --- | --- | --- | --- | --- | --- | --- | --- | --- | --- | --- | --- | --- |
| C14:0 |  | 0.15 | -0.19 | 0.18 | -0.17 | -0.01 | 0.47 | 0.20 | 0.48 | 0.32 | -0.64 | 0.20 | 0.17 | -0.20 | -0.38 | 0.11 | -0.69 | 0.10 | -0.50 | -0.08 | 0.02 |
| C16:0 | 0.22 |  | -0.07 | -0.10 | -0.12 | -0.09 | 0.07 | -0.10 | 0.11 | -0.11 | -0.08 | -0.03 | -0.07 | -0.05 | -0.06 | -0.06 | -0.13 | -0.07 | -0.04 | -0.12 | -0.03 |
| C18:0 | -0.14 | 0.13 |  | -0.11 | 0.02 | -0.13 | -0.42 | -0.31 | -0.28 | -0.25 | -0.08 | -0.31 | -0.19 | -0.12 | -0.15 | -0.20 | -0.04 | -0.18 | -0.07 | -0.20 | -0.18 |
| C20:0 | 0.18 | -0.17 | -0.24 |  | 0.49 | 0.65 | 0.56 | 0.03 | -0.36 | 0.87 | 0.06 | 0.75 | 0.96 | 0.69 | 0.39 | 0.92 | 0.07 | 0.87 | 0.07 | 0.80 | 0.70 |
| C22:0 | -0.28 | -0.17 | -0.08 | 0.3 |  | 0.57 | 0.29 | 0.01 | -0.65 | 0.29 | 0.50 | 0.35 | 0.47 | 0.55 | 0.48 | 0.52 | 0.49 | 0.48 | 0.61 | 0.65 | 0.48 |
| C24:0 | -0.06 | -0.22 | -0.28 | 0.63 | 0.37 |  | 0.42 | -0.01 | -0.50 | 0.49 | 0.29 | 0.46 | 0.71 | 0.53 | 0.39 | 0.71 | 0.29 | 0.58 | 0.29 | 0.85 | 0.81 |
| C14:1 cis-9 | 0.51 | 0.05 | -0.49 | 0.48 | 0.09 | 0.35 |  | 0.16 | -0.06 | 0.54 | -0.01 | 0.49 | 0.61 | 0.31 | 0.14 | 0.57 | -0.08 | 0.51 | 0.03 | 0.48 | 0.46 |
| C16:1 cis-9 | 0.23 | -0.2 | -0.35 | 0.08 | -0.06 | 0.03 | 0.25 |  | 0.18 | 0.12 | -0.13 | 0.30 | 0.01 | 0.14 | -0.04 | 0.01 | -0.15 | 0.01 | -0.01 | 0.05 | 0.01 |
| C18:1 cis-9 | 0.53 | 0.15 | -0.16 | -0.21 | -0.6 | -0.37 | 0.08 | 0.3 |  | -0.03 | -0.83 | -0.16 | -0.37 | -0.63 | -0.64 | -0.43 | -0.80 | -0.38 | -0.74 | -0.60 | -0.45 |
| C20:1 cis-11 | 0.32 | -0.1 | -0.35 | 0.89 | 0.19 | 0.5 | 0.55 | 0.17 | 0.06 |  | -0.20 | 0.76 | 0.84 | 0.52 | 0.18 | 0.79 | -0.19 | 0.75 | -0.17 | 0.61 | 0.54 |
| C18:2 n-6 | -0.68 | -0.22 | -0.14 | -0.08 | 0.53 | 0.23 | -0.14 | -0.22 | -0.82 | -0.25 |  | -0.02 | 0.11 | 0.47 | 0.65 | 0.19 | 0.92 | 0.17 | 0.83 | 0.40 | 0.26 |
| C18:2 t-9c-11 | 0.19 | -0.15 | -0.33 | 0.74 | 0.22 | 0.38 | 0.43 | 0.24 | -0.08 | 0.71 | -0.1 |  | 0.71 | 0.65 | 0.35 | 0.69 | -0.04 | 0.63 | 0.05 | 0.63 | 0.51 |
| C18:2 t-12c-10 | 0.19 | -0.18 | -0.29 | 0.95 | 0.31 | 0.68 | 0.56 | 0.06 | -0.25 | 0.86 | -0.03 | 0.73 |  | 0.67 | 0.39 | 0.96 | 0.10 | 0.84 | 0.10 | 0.82 | 0.75 |
| C18:3 n-6 | -0.24 | -0.24 | -0.23 | 0.66 | 0.51 | 0.44 | 0.19 | 0.13 | -0.48 | 0.55 | 0.33 | 0.64 | 0.61 |  | 0.61 | 0.68 | 0.44 | 0.60 | 0.50 | 0.73 | 0.59 |
| C18:3 n-3 | -0.43 | -0.16 | -0.2 | 0.26 | 0.49 | 0.29 | -0.03 | -0.14 | -0.59 | 0.14 | 0.65 | 0.28 | 0.25 | 0.52 |  | 0.43 | 0.58 | 0.40 | 0.60 | 0.52 | 0.39 |
| C20:2 n-6 | 0.1 | -0.22 | -0.27 | 0.92 | 0.35 | 0.68 | 0.46 | 0.01 | -0.32 | 0.81 | 0.05 | 0.67 | 0.95 | 0.61 | 0.27 |  | 0.18 | 0.83 | 0.20 | 0.84 | 0.75 |
| C20:3 n-3 | -0.68 | -0.22 | -0.16 | 0.02 | 0.55 | 0.27 | -0.16 | -0.22 | -0.8 | -0.18 | 0.9 | -0.03 | 0.04 | 0.43 | 0.6 | 0.15 |  | 0.17 | 0.80 | 0.41 | 0.23 |
| C20:4 n-6 | 0.13 | -0.21 | -0.26 | 0.86 | 0.37 | 0.57 | 0.46 | 0.04 | -0.27 | 0.79 | 0.05 | 0.62 | 0.83 | 0.54 | 0.29 | 0.83 | 0.12 |  | 0.19 | 0.72 | 0.61 |
| C20:5 n-3 | -0.58 | -0.17 | -0.15 | -0.13 | 0.61 | 0.14 | -0.16 | -0.16 | -0.67 | -0.26 | 0.81 | -0.11 | -0.11 | 0.3 | 0.55 | 0 | 0.81 | 0.05 |  | 0.41 | 0.27 |
| C22:5 n-3 | -0.13 | -0.27 | -0.34 | 0.79 | 0.53 | 0.81 | 0.36 | 0.03 | -0.51 | 0.62 | 0.32 | 0.58 | 0.79 | 0.68 | 0.44 | 0.83 | 0.41 | 0.71 | 0.28 |  | 0.83 |
| C22:6 n-3 | -0.05 | -0.18 | -0.24 | 0.66 | 0.32 | 0.83 | 0.35 | -0.01 | -0.35 | 0.54 | 0.2 | 0.4 | 0.68 | 0.44 | 0.27 | 0.71 | 0.22 | 0.57 | 0.11 | 0.82 |  |

^a^The concentrations of fatty acids were expressed as a percentage of total fatty acid methyl esters quantified.
